# Impact of Leukapheresis and Biological Risk Markers on Early Mortality in Patients with Hyperleukocytic Acute Myeloid Leukemia

**DOI:** 10.3390/medicina62010035

**Published:** 2025-12-24

**Authors:** Mirjana Čučaković, Lazar Trajković, Marija Dinić, Nikola Pantić, Nikica Sabljić, Zlatko Pravdić, Jovan Rajić, Violeta Milošević, Mirjana Mitrović, Ana Vidović, Nada Suvajdžić-Vuković, Andrija Bogdanović, Ljubomir Jaković, Marijana Virijević

**Affiliations:** 1 Clinic of Hematology, University Clinical Centre of Serbia, 11000 Belgrade, Serbia; 2 Department for Therapeutic Apheresis Procedures and Intraoperative Blood Salvage, University Clinical Center of Serbia, 11000 Belgrade, Serbia; 3 Faculty of Medicine, University of Belgrade, 11000 Belgrade, Serbia

**Keywords:** AML, hyperleukocytosis, leukapheresis, risk markers, early mortality

## Abstract

*Background and Objectives*: Hyperleukocytosis in acute myeloid leukemia (AML) is life-threatening, often complicated by leukostasis, tumor lysis syndrome (TLS), and disseminated intravascular coagulation (DIC), with very high early mortality. Leukapheresis (LA) can rapidly reduce circulating blast burden, but its effect on survival and prognostic relevance of disease markers remains unclear. *Materials and Methods*: We retrospectively analyzed 74 adult AML patients with WBC > 100 × 10^9^/L treated at the University Clinical Center of Serbia between 2014 and 2024: 28 received LA plus cytoreduction (LA group), and 46 received cytoreduction alone (non-LA group). We evaluated 15-, 30-, and 90-day mortality and overall survival (OS), and assessed clinical, laboratory, and immunophenotypic predictors using Cox regression, with separate subgroup analyses. *Results*: Patients in the LA group had significantly higher baseline leukocyte counts and LDH (*p* = 0.18 and *p* = 0.024, respectively). Although LA resulted in a median 34% reduction in WBC, there was no statistically significant difference in early mortality: 15-day survival was 68% vs. 76% (HR 0.70, *p* = 0.423), 30-day survival 50% vs. 65% (HR 0.62, *p* = 0.197), and 90-day survival 39.3% vs. 41.3% (HR 0.85, *p* = 0.604). Median OS was similarly poor, about 1 month in the LA group compared to 2 months in the non-LA (HR 0.73). Across all patients, ECOG PS ≥2, elevated LDH, TLS, and DIC were the strongest indicators of early death. In the LA group, elevated LDH and increased peripheral blood (PB) monocyte count predicted 15-day mortality (*p* = 0.021 and *p* = 0.031, respectively), but lost significance by day 90. In non-LA patients, CD25 positivity (*p* = 0.034) and DIC (*p* = 0.045) predicted 15-day death. By day 90, CD25 expression (*p* = 0.048) remained prognostic, while PB blast percentage (*p* = 0.045) and PB monocyte count (*p* = 0.017) emerged as additional adverse prognostic predictors in the non-LA group. In multivariate analysis, higher PB blast percentage, CD25 positivity, and ECOG PS ≥ 2 independently predicted poorer OS. *Conclusions*: Although LA did not reduce early mortality in the entire cohort, the loss of prognostic significance of elevated LDH, high PB blast percentage, PB monocyte burden, and CD25 expression in the LA group may suggest that the intervention can attenuate the impact of biologically aggressive disease.

## 1. Introduction

Acute myeloid leukemia (AML) is a clonal disorder characterized by uncontrolled proliferation of hematopoietic stem cells, leading to bone marrow failure and the accumulation of immature, nonfunctional myeloid cells in the bone marrow (BM) and other organs [1,2]. This uncontrolled proliferation of myeloid progenitor cells can result in significant leukocytosis in 5–20% of the patients with newly diagnosed AML [3,4,5,6]. Hyperleukocytosis (HL), commonly defined by a white blood cell (WBC) count > 100 × 10^9^/L, presents a critical challenge in the effective management of new-onset AML, due to a greater risk of subsequent leukostasis, disseminated intravascular coagulation (DIC), and tumor lysis syndrome (TLS) [6,7,8].

An elevated WBC count is usually indicative of high cell turnover and correlates with the severity of TLS, which, as a result of hypermetabolic leukemic cell breakdown, can lead to metabolic disturbances, renal failure, cardiac arrhythmias, and fatal outcome [4,9]. Approximately 10–30% of patients presenting with HL develop DIC, characterized by systemic intravascular activation of coagulation, which can lead to multiorgan dysfunction, thrombosis, and/or excessive bleeding [9]. It is caused by rapid cell turnover and high levels of released tissue factor, which subsequently activates extrinsic pathway [4,8].

The most common cause of morbidity and mortality is leukostasis, which is present in almost half of the patients with a WBC count > 100 × 10^9^/L [9]. The development of leukostasis is explained by the disturbance of microcirculation caused by increased blood viscosity due to elevated WBC count, together with reduced deformability of myeloid blasts [8,10,11]. However, since leukostasis can appear even at a significantly lower WBC count than the mentioned threshold, it is suggested that interactions between blasts and endothelial cells have an important role in leukostasis pathogenesis [12,13]. Blast cells secrete cytokines (TNF-β and IL-1β), which activate endothelial cells (mediated by adhesion receptors), leading to blast cell adhesion to vascular endothelium and chemokine-guided migration into the interstitial space [8,13]. Clinically, leukostasis most commonly affects the lungs, central nervous system, and kidneys [4,12]. It is a life-threatening condition, with data showing that the early mortality rate can be up to 50% [8]. Therefore, given the dramatic effects, HL and leukostasis present a hematologic emergency that requires immediate intervention.

Cytoreduction by chemotherapy (i.e., hydroxyurea, low-dose cytarabine) and leukapheresis (LA) are common strategies for achieving rapid WBC reduction. LA is a strategy for immediate reduction of an excessive WBC by mechanical separation, and a single procedure can reduce the WBC burden by up to 70% [14]. The procedure, by mechanical removal of cell mass, reduces leukostatic manifestations caused by the obstruction of small vessels. Also, LA can increase the fraction of leukemia cells in S-phase, which may increase the efficiency of concomitant S-phase specific agents, like cytarabine [14,15]. However, since the majority of blasts are still located in the BM, there is often a quick rebound after the procedure [11].

Even with the remarkable initial improvement in symptomatic disease, there are no standardized guidelines that define the role of LA. In everyday practice, most clinicians use American Society for Apheresis (ASFA) guidelines, where LA is accepted as second-line therapy [16]. The main reason why the use of LA in hyperleukocytic adult AML is still under debate lies in the fact that its effects on early outcomes are inconsistent in the literature. Several studies have reported LA to reduce early mortality in hyperleukocytic AML [17,18,19,20]. However, other studies did not show any benefit from performing emergency LA [21,22,23,24]. Therefore, the best approach to management of HL still remains unclear, and it is regulated according to Institutional protocols.

The aim of our study was to determine the clinical effect of LA by evaluating 15-day, 30-day, and 90-day mortality, as well as overall survival (OS) in AML patients with HL.

## 2. Materials and Methods

A retrospective study included 74 consecutive newly diagnosed adult (≥18 years) AML patients, with hyperleukocytosis, defined as WBC count > 100 × 10^9^/L, diagnosed and treated at University Clinical Center of Serbia between January 2014 and December 2024. They were identified from a cohort of 575 consecutive adults with AML (74/575; 12.8%), seen at the same period. Patients with acute promyelocytic leukemia were not included in the study. AML diagnosis was established according to World Health Organization (WHO) 2016 recommendations [25], based on cytomorphological, immunophenotypical, molecular, and cytogenetic studies. Risk stratification (favorable, intermediate, adverse) of AML patients was carried out in keeping with European LeukemiaNet (ELN) 2017 recommendations [26]. When establishing the diagnosis, the overall performance status of the patients was determined according to Eastern Cooperative Oncology Group performance status (ECOG PS) [27], and the significance of existing comorbidities was determined on the basis of the hematopoietic cell transplantation specific comorbidity index (HCT-CI) score [28]. Extramedullary disease (EMD) was defined as hepatosplenomegaly, lymphadenopathy, gingival hypertrophy, or neuroleukemia.

Evaluated laboratory parameters were WBC, hemoglobin (Hb), platelet (Plt) count, lactate dehydrogenase (LDH), peripheral blood (PB) monocyte count, BM, and PB blast percentage. Also, the presence of overt DIC, assessed according to the 2018 revision of the International Society on Thrombosis and Haemostasis (ISTH) DIC score [29], TLS [30], and acute kidney injury (AKI) [31], was noted at the time of diagnosis.

The patients were divided in two groups based on the initial therapeutic approach (LA vs. non-LA). The decision to perform emergency LA was based on the presence of leukostasis, according to Institutional protocols. All patients received cytoreductive therapy (hydroxyurea or cytarabine). Hydroxyurea alone was initiated at a dose of 50 mg/kg/day in 69/74 (93.2%) patients, and cytarabine was administered to hydroxyurea-resistant patients (5/74, 6.75%) at a dose of 1 g/day. After cytoreduction, patients received intensive (7 + 3 induction, followed by intermediate-dose cytarabine (IDAC) consolidation and allogenic hematopoietic stem-cell transplantation) or non-intensive chemotherapy (low-dose chemotherapy, azacitidin, venetoclax) or supportive care, depending on the general performance status and the comorbidity index. Patients with FLT3 mutation who were candidates for intensive therapy additionally received the tyrosine-kinase inhibitor midostaurin. Early death was defined as death from any cause within the first 30 days from AML diagnosis and commencing cytoreductive therapy [7].

The leukapheresis procedure was performed using a continuous-flow blood cell separator Spectra Optia^®^ (SPO, Terumo BCT, Lakewood, CO, USA). During each LA session, the target processed blood volume was approximately 1.5–2.0 times the patient’s total circulating blood volume, adjusted according to hemodynamic stability and baseline WBC count, with an average procedure duration of 4 h. Acid citrate dextrose solution A (ACD-A) was used as an anticoagulant, with a blood-to-citrate ratio of 12:1 to 20:1, and with the concomitant administration of intravenous calcium gluconate.

The study was performed in accordance with the Declaration of Helsinki after approval by the Institutional Review Board (protocol number III 41004). General informed consent was obtained from all individual participants included in the study. 

All statistical analysis was performed using SPSS version 26 statistical software (Chicago, IL, USA). Categorical variables were expressed as absolute or relative frequencies, and continuous variables were summarized as mean and the standard deviation (SD), and median and range, depending on the distribution status. Differences between groups were assessed using the Chi-square or Fisher’s exact test for categorical variables and the Mann–Whitney U test for continuous variables, as appropriate. Univariate analysis using the Cox regression model was performed in order to identify potential variables with significant correlations with the mortality rate and overall survival (OS). The independent association of evaluated biomarkers with early mortality and OS was investigated by multivariate analysis using the Cox proportional hazards regression model. For survival analysis, the Kaplan–Meier method and log-rank test to compare survival curves between groups were applied. The significance level was set at *p* < 0.05.

## 3. Results

### 3.1. Patient-Related and Leukemia-Related Characteristics

The median age of patients in the study cohort was 55 years (range, 18–81 years), with overall male predominance (M:F = 1.17). Cytoreduction with hydroxyurea or cytarabine as well as therapeutic LA was conducted in 28/74 (37.8%) patients (LA group), whereas 46/74 (62.2%) patients received cytoreduction alone (non-LA group). LA was performed in patients with symptomatic disease. The majority of patients showed signs of pulmonary leukostasis (53.6%), followed by neurological manifestations (32.1%) and AKI (14.3%). The baseline patients’ characteristics at AML diagnosis are listed in Table 1. Patients who underwent LA had significantly higher WBC counts (median 194 × 10^9^/L, range: 103–470), compared to the non-LA group (median 141 × 10^9^/L, range: 100.2–473.2), *p* = 0.018. The average number of LA procedures per patient was 2.7, and the median WBC count after first LA was 118 × 10^9^/L (range: 42–366), with an average achieved WBC reduction of 34.5% (range: 8–67). We found no statistical difference between the cohorts regarding age (LA: median 50 years, range: 19–80; non-LA: median 58 years, range 18–81; *p* = 0.198), sex (male/female ratio in LA: 1; non-LA: 1.3; *p* = 0.585), as well as initial poor performance status (LA: 32.1%; non-LA: 36.9%; *p* = 0.674). The non-LA group had a higher comorbidity index (HCT CI ≥ 2 in LA: 21.4%; non-LA: 41.3%), but it did not reach statistical significance (*p* = 0.08). Almost half of the patients (36/74, 48.6%) had no comorbidities, 25/74 (33.8%) patients had one comorbidity, and 13/74 (17.6%) patients had two or more comorbidities. The presence of comorbidities (none, one, two or more) did not differ between the LA and non-LA groups (*p* > 0.05), as shown in Table 1. Underlying cardiovascular disease (hypertension, cardiomyopathy, previous myocardial infarction, arrhythmias, valvular disease) was present in 28/74 (37.8%) patients, endocrine and metabolic disease (diabetes mellitus, hyperlipidemia, obesity) was present in 11/74 (14.9%) patients, pulmonary disease (asthma, chronic pulmonary obstructive disease) in 7/74 (9.5%) patients, autoimmune disease (thyroid dysfunction, rheumatoid arthritis) in 4/74 (5.4%) patients, and 1/74 (1.3%) patient had chronic kidney disease. There was no statistical difference between cohorts regarding the presence of cardiovascular comorbidity (LA: 35.7% vs. non-LA: 39.1%, *p* = 0.769).

Patients in both groups presented with thrombocytopenia (LA: median 45.5 × 10^9^/L, range: 11–284, non-LA: median 61 × 10^9^/L, range: 14–244; *p* = 0.107) and anemia (LA: median 95 g/L, range 30–138; non-LA: median 96 g/L, range 41–140; *p* = 0.369). Patients in the LA group had significantly higher LDH (median 1802 U/L, range 324–8505) compared to the non-LA group (1164 U/L, range 219–6680), *p* = 0.024. There was no difference regarding PB blasts (LA: median 85%, range 6–100; non-LA: median 71%, range 0–98; *p* = 0.477), BM blasts (LA: median 79.5%, range 28–96, non-LA: median 73.5%, range 20–92; *p* = 0.409), and PB monocytes (LA: median 3, range 0–88; non-LA: median 4, range 0–86; *p* = 1.00). EMD was present in 18 (64.3%) patients in the LA group and 32 (69.6%) patients in the non-LA group (*p* = 0.638).

Molecular and genetic features did not significantly differ between the two cohorts. Unfavorable cytogenetic was present in 10.7% of patients in the LA cohort and in 13% of patients in the non-LA cohort. Also, there was no significant difference regarding the presence of NPM1 mutation (25% vs. 17.4%; *p* = 0.230), FLT3 mutation (39.3% vs. 30.4%; *p* = 0.302), CD56 positivity (35.7% vs. 36.9%; *p* = 0.911), CD117 positivity (75% vs. 82.6%; *p* = 0.715), CD7 positivity (32.2% vs. 28.3%; *p* = 0.670), CD25 positivity (42.9% vs. 34.8%; *p*= 0.712), and CD11c positivity (67.9% vs. 76.1%; *p* = 0.283). The criteria for overt DIC were met by 18/28 (64.3%) patients in the LA group and 27/46 (58.7%) patients in the non-LA group (*p* = 0.633). On the other hand, fewer patients in both groups presented with signs and symptoms of TLS (LA: 14.3% vs. non-LA: 13%; *p* = 1.00). The presence of AKI at the time of diagnosis was noted in 8/28 (28.6%) patients in the LA group and 11/46 (23.9%) patients in the non-LA group (*p* = 0.565). Detailed cytogenetic and molecular genetic characteristics and aberration are listed in Table 1. After initial cytoreduction, the majority of patients were treated with intensive induction therapy (LA: 64.3% vs. non-LA: 56.5%; *p* = 0.509) and 13/25 (52%) received tyrosine-kinase inhibitor. Median time from admission to induction therapy initiation was 3 days (range 2–5).

### 3.2. Early Mortality Analysis (15-, 30-, 90-Day)

**Figure 1:**
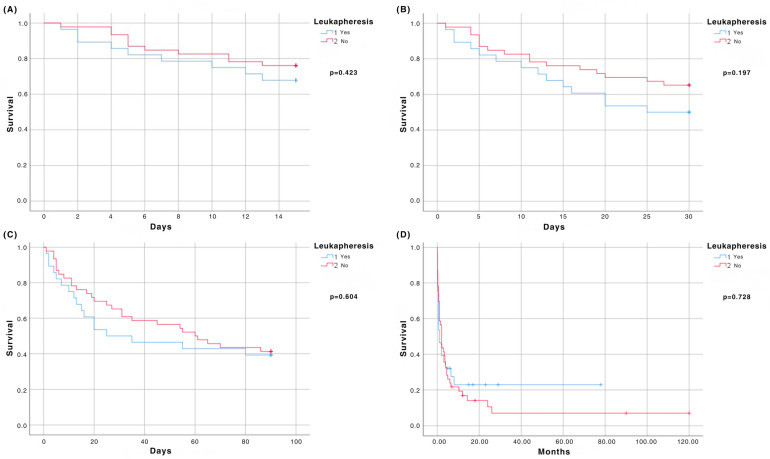
(**A**) Survival at 15 days; (**B**) Survival at 30 days; (**C**) Survival at 90 days; (**D**) Overall survival.

We observed no significant difference regarding early mortality in the LA and non-LA groups (Figure 1). The 15-day mortality rates were 32.1% and 23.9%, respectively (HR 0.698; 95% CI 0.289–1.684; *p* = 0.423). The 30-day mortality was 50% in the LA and 34.8% in the non-LA group (HR 0.623; 95% CI 0.304–1.277; *p* = 0.197), whereas the 90-day mortality was 60.7% and 58.7%, respectively (HR 0.851; 95% CI 0.464–1.563; *p* = 0.604) (Figure 1). Factors associated with early mortality in patients with HL are summarized in Table 2.

At 15 days, overall mortality was predominantly influenced by poor performance status (ECOG PS ≥ 2; *p* < 0.01), elevated LDH (*p* < 0.01), AKI (*p* < 0.01) and the presence of TLS and DIC (both *p* = 0.01), and receipt of intensive induction therapy (*p* < 0.01). ECOG PS ≥ 2, elevated LDH and AKI, and intensive induction chemotherapy remained significant predictors in both LA and non-LA subgroups. TLS was particularly relevant among LA patients (*p* = 0.033), whereas DIC showed significance in the non-LA subgroup (*p* = 0.045). Higher monocyte count was significant only in the LA group (*p* = 0.031). Regarding immunophenotype, CD25 expression did not demonstrate an association (*p* = 0.067) in the overall cohort, but reached statistical significance in the non-LA subgroup (*p* = 0.034).

At 30 days, ECOG PS ≥ 2 (*p* < 0.01), elevated LDH (*p* < 0.01), DIC (*p* = 0.018), TLS (*p* = 0.002), AKI (*p* = 0.001), and intensive induction therapy (*p* < 0.01) remained the principal factors influencing mortality, together with higher WBC count (*p* = 0.032). TLS retained strong significance in the LA subgroup (*p* = 0.004), whereas LDH remained significant in the non-LA subgroup (*p* < 0.01). Although PB blast percentage was not significant overall, it reached *p* = 0.021 in non-LA patients.

By 90 days, overall mortality continued to be influenced by ECOG PS ≥ 2 (*p* < 0.01), elevated LDH (*p* < 0.01), TLS (*p* = 0.017), AKI (*p* = 0.015), and intensive induction therapy (*p* < 0.01). Again, TLS retained significance only in the LA group (*p* = 0.004), and high LDH in the non-LA group (*p* < 0.01). In the non-LA subgroup, PB blast percentage (*p* = 0.014), CD25 expression (*p* = 0.048), and PB monocytes (*p* = 0.017) emerged as additional predictors.

No significant associations were observed for age, HCT-CI, presence of ≥2 comorbidities, presence of cardiovascular comorbidity, Plt, Hb, BM blast percentage, other immunophenotypical markers (CD7, CD11c, CD56, CD117), or cytogenetic/molecular features (FLT3, NPM1, or unfavorable karyotype).

Further analysis showed that there was no statistical significance regarding the impact of LA on patients who subsequently received intensive vs. non-intensive induction therapy at all time points: 15-day mortality (HR 1.590; 95% CI 0.119–21.157; *p* = 0.725), 30-day mortality (HR 1.727; 95% CI 0.369–8.081; *p* = 0.488), 90-day mortality (HR 0.661; 95% CI 0.195–2.238; *p* = 0.506). Logistic regression showed that LA was not associated with an increased likelihood of developing AKI (OR 1.27; 95% CI 0.44–3.69; *p* = 0.657).

Infectious complications (sepsis, pneumonia, neutropenic enterocolitis) were the leading cause of death (LA: 8/28 (28.6%); non-LA: 24/46 (52.2%); overall 32/74 (43.2%)), followed by metabolic complications (TLS, DIC, renal failure) in 16.2% (12/74) of the patients (LA: 4/28 (14.3%); non-LA: 8/46 (17.4%)), cardiovascular complications (cardiopulmonary collapse, arrhythmias) in 14.9% (11/74) of the patients (LA: 2/28 (7.1%); non-LA: 9/46 (19.6%)) and leukostasis-related (respiratory and neurologic failure) complications in 8.1% (6/74) of the patients (LA: 6/28 (21.4%); non-LA: 0/46).

### 3.3. Overall Survival

Median OS was short in both cohorts, and the difference was not statistically significant (LA: 1 month; 95% CI 0.0–2.4 vs. non-LA: 2 months; 95% CI 1.2–28; *p* = 0.728). Initial performance status (ECOG PS) ≥ 2 (*p* < 0.01), higher WBC count (*p* = 0.017), and PB blast percentage (*p* = 0.024), as well as elevated LDH (*p* = 0.002), presence of TLS (*p* = 0.028), AKI (*p* = 0.011), CD7 (*p* = 0.044), and CD25 (*p* = 0.018) positivity were found to be associated with shorter OS. Conversely, patients who received intensive induction therapy (*p* < 0.01) and underwent allo-SCT (*p* = 0.012) had significantly longer OS. Other evaluated parameters had no impact on OS. In the multivariate Cox regression model (Table 3), higher PB blast percentage (HR 1.017; 95% CI 1.005–1.029; *p* = 0.004), CD25 positivity (HR 2.075; 95% CI 1.152–3.738; *p* = 0.015), and ECOG PS ≥ 2 (HR 4.588; 95% CI 1.675–12.569; *p* = 0.03) were identified as independent unfavorable factors for OS. Further analysis showed that LA itself had no influence on different OS within the subgroups of patients who received intensive vs. non-intensive induction therapy (HR 0.462; 95% CI 0.159–1.340; *p* = 0.155).

## 4. Discussion

In this retrospective study of newly diagnosed hyperleukocytic AML patients, despite the higher baseline WBC count and leukostasis symptoms in the LA cohort, no significant difference was observed in 15-, 30- and 90-day mortality between the two groups. Studies by Kuo [23], Choi et al. [22], Martínez-Cuadrón et al. [24], and Rinaldi et al. [21] demonstrated that LA does not have an impact on short-term outcomes. In contrast, studies by Bug et al. [17], Nan et al. [18], Lee et al. [19], and Stahl et al. [20] reported reduced early mortality in patients who underwent LA. A comprehensive meta-analysis by Bewersdorf et al. [8] showed no significant short-term benefit from LA, arguing for its routine use. Conflicting results across published cohorts are likely due to patient selection, timing of LA initiation, and supportive care. In our cohort, LA was performed in patients with overt leukostasis manifestations, primarily pulmonary and neurological, suggesting a selection of more critically ill patients. Therefore, similar mortality rates between the two groups might suggest that LA provided temporary hemodynamic and microcirculatory stabilization that compensated for the initial risk in patients who underwent the procedure, but it might not be sufficient to prevent early death.

Our analysis identified several clinical and biological factors associated with early mortality, including poor performance status (ECOG PS ≥ 2) and the presence of TLS, AKI, and DIC. These variables remained significant across both LA and non-LA subgroups, emphasizing that early death in hyperleukocytic AML is primarily determined by disease aggressiveness and systemic metabolic dysfunction, rather than the WBC count itself. Among laboratory findings, elevated LDH, high PB blast percentage, PB monocyte count, and CD25 expression emerged as additional unfavorable prognostic factors at 90 days. Neither BM blast percentage, karyotype, FLT3, nor NPM1 mutations had an impact on short-term survival, underscoring that early mortality is predominantly a result of clinical risk factors [31]. Importantly, intensive induction therapy was associated with significantly reduced mortality at all three time points across both LA and non-LA subgroups, indicating its protective effect even in the presence of high-risk clinical features [32,33].

Our observations support the idea that certain subgroups might benefit from the LA procedure. The advantage of LA was reported in several studies showing that patients with HL and symptomatic leukostasis may experience short-term relief of respiratory and neurological manifestations following the cytoreduction by LA [6,34]. This effect is based on the rapid mechanical removal of circulating blasts and the possibility that repeated procedures can mobilize marginated leukemic cells into the bloodstream, thereby dissolving WBC aggregates that have formed in small vessels [34,35]. The association of high PB blast percentage, PB monocyte count, and CD25 expression with increased 90-day mortality suggests that leukemias with a high proliferative index and cytokine production are biologically aggressive. Expression of CD25, the α-chain of the interleukin-2 (IL-2) receptor, is associated with adverse outcomes, and its expression has been associated with a higher WBC count, reduced response to induction therapy, and the presence of FLT3-ITD, a known adverse prognostic factor, suggesting it may function as a surrogate marker for the mutation [36,37,38]. Farid et al. [39] proposed that a subgroup of AML patients with HL, leukostasis, and FLT3-ITD mutation have benefit from early LA. The studies have also shown that CD25 expression was associated with adverse outcome in both intermediate and high-risk cytogenetic groups, indicating that CD25 serves as an independent prognostic factor in the majority of AML patients [36].

Although LA did not reduce early mortality in the entire cohort, the loss of prognostic significance of elevated LDH, high PB blast percentage, and CD25 expression in the LA group may suggest that the intervention can attenuate the impact of biologically aggressive disease. These markers reflect high proliferative activity and metabolic turnover as well as leukostasis risk—all of which LA might temporarily alleviate by rapidly reducing circulating blasts, effectively decreasing tumor burden, and reducing blood viscosity. Consequently, while LA cannot modify the underlying disease biology, it may temporarily stabilize patients with hyperproliferative or CD25-positive AML, allowing intensive induction therapy initiation. These findings indicate that the PB blast burden and CD25 expression represent not only indicators of biologically aggressive disease, but may also additionally serve as practical tools for therapeutic risk stratification in hyperleukocytic AML. By identifying patients with highly proliferative, cytokine-driven disease, who are at higher risk for early deterioration, these biomarkers could help refine early management algorithms regarding the selective use of LA.

In addition to PB blast burden and CD25 expression, our exploratory analysis demonstrated that PB monocyte counts may also contribute to adverse early outcomes, although with a distinct pattern between LA and non-LA groups. Monocytes and monocytic blasts are particularly likely to provoke leukostasis due to larger size, reduced deformability, and tendency to adhere strongly to the endothelium, which explains why AML with monocytic differentiation (M4/M5) is frequently associated with severe HL and leukostasis [6,8,12,17]. In our cohort, elevated PB monocyte counts correlated with higher 15-day mortality among patients who received LA. These findings suggest that LA might be insufficient to reverse the microvascular damage that has already occurred in highly aggressive disease. Conversely, PB monocyte counts predicted 90-day mortality only in patients who did not undergo LA, which indicated that LA might mitigate the long-term consequences of monocytic disease by rapidly reducing the circulating blast and monocyte burden and improving viscosity. These observations, although exploratory, highlight the need for future analysis to identify which patient subgroups might benefit from early LA.

There are several limitations of our study. First, the retrospective nature of this study limits the ability to establish causal relationships and introduces the risk of unrecognized confounders. Second, selection bias is present because the decision to perform LA was determined by clinical judgment, primarily based on the presence of leukostasis manifestations. As a result, patients undergoing LA may have presented a clinically more severe subgroup at diagnosis. Furthermore, the data regarding the time from clinical onset of AML to referral to our Center were not uniformly recorded, which could have influenced survival outcomes. Certain clinical variables, such as the severity of organ dysfunction, detailed metabolic parameters, and dynamic laboratory changes during cytoreduction, were not uniformly available for all patients. These missing data and relatively small sample size may have limited the precision of multivariable models and reduced the ability to detect meaningful subgroup effects. Nonetheless, this study is one of the few single-center analyses integrating clinical, cytogenetic, and immunophenotypic data in the evaluation of LA efficacy.

## 5. Conclusions

Our findings demonstrate that LA was not shown to improve early mortality or OS in unselected patients with hyperleukocytic AML. The strongest predictors for early death were poor performance status, metabolic complications, and high proliferative activity indices, such as elevated LDH, PB blast burden, and, in certain analyses, PB monocyte burden. Importantly, the prognostic significance of PB blast percentage and CD25 positivity was attenuated among patients who underwent LA, suggesting that LA may mitigate adverse effects of highly proliferative, cytokine-driven disease. Although the procedure did not demonstrate an overall benefit, these observations support the idea that LA may preserve a role as an emergency cytoreductive strategy for patients with symptomatic leukostasis, rapidly escalating leukocytosis, or evolving TLS, particularly when chemotherapy cannot be initiated immediately. Future prospective studies should focus on refining patient selection and integrating biologic markers, such as CD25 expression, PB blast kinetics, and monocyte burden, in order to determine the subgroup of patients with HL who might benefit from this procedure.

## Figures and Tables

**Table 1:** Patients’ and leukemia-related characteristics at acute myeloid leukemia (AML) diagnosis.

	All HL *n* = 74	LA *n* = 28	Non-LA *n* = 46	*p* Value
Age (median, range)	55 (18–81)	50 (19–80)	58.5 (18–81)	0.198
Age > 60 years (*n*, %)				
Yes	28 (37.8)	8 (28.6)	20 (43.5)	0.200
No	46 (62.2)	20 (71.4)	26 (56.5)	
Sex (*n*, %)				
Male	40 (54.1)	14 (50)	26 (56.5)	0.585
Female	34 (45.9)	14 (50)	20 (43.5)	
M:F ratio	1.17	1	1.3	
ECOG PS ≥ 2				
Yes	26 (35.1)	9 (32.1)	17 (36.9)	0.674
No	48 (64.9)	19 (67.9)	29 (63.1)	
HCT-CI (*n*,%)				
Low (0–2)	49 (66.2)	22 (78.6)	27 (58.7)	0.08
High (>2)	25 (33.8)	6 (21.4)	19 (41.3)	
Comorbidities (*n*, %)				
0	36 (48.6)	15 (53.6)	21 (45.6)	0.584
1	25 (33.8)	9 (32.1)	16 (34.8)	0.816
≥2	13 (17.6)	4 (14.3)	9 (19.6)	0.522
CV comorbidity (*n*, %)				
Yes	28 (37.8)	10 (35.7)	18 (39.1)	0.769
No	46 (62.2%)	18 (64.3)	28 (60.9)	
WBC (×10^9^/L) (median, range)	161 (100.2–473.2)	194 (103–470)	141 (100.2–473.2)	**0.018**
Hemoglobin (g/L) (median, range)	95.5 (30–140)	95 (30–138)	96 (41–140)	0.369
Platelet count (×10^9^/L) (median, range)	54 (11–284)	45.5 (11–284)	61 (14–244)	0.107
LDH (U/L) (median, range)	1454.4 (219–8505)	1802 (324–8505)	1164 (219–6680)	**0.024**
Monocytes PB (median, range)	4 (0–88)	3 (0–88)	4 (0–86)	1.00
PB blasts (median %, range)	78 (0–100)	85 (6–100)	71 (0–98)	0.477
BM blasts (median %, range)	75 (20–96)	79.5 (28–96)	73.5 (20–92)	0.409
Extramedullary disease (*n*, %)				
Yes	50 (67.6)	18 (64.3)	32 (69.6)	0.638
No	24 (32.4)	10 (35.7)	14 (30.4)	
Cytogenetics (*n*, %)				
Favorable and intermediate	55 (74.3)	20 (71.4)	35 (76.1)	1.00
Unfavorable	9 (12.2)	3 (10.7)	6 (13)	
Missing	10 (13.5)	5 (17.9)	5 (10.9)	
NPM1 (*n*,%)				
Mutated	15 (20.3)	7 (25)	8 (17.4)	0.230
Wild-type	21 (28.4)	14 (50)	7 (15.2)	
Missing	38 (51.3)	7 (25)	31 (67.4)	
FLT3 (*n*,%)				
Mutated	25 (33.8)	11 (39.3)	14 (30.4)	0.302
Wild-type	22 (29.7)	13 (46.4)	9 (19.6)	
Missing	27 (36.5)	4 (14.3)	23 (50)	
CD56 (*n*,%)				
Positive	27 (36.5)	10 (35.7)	17 (36.9)	0.911
Negative	42 (56.7)	15 (53.6)	27 (58.7)	
Missing	5 (6.8)	3 (10.7)	2 (4.4)	
CD117 (*n*,%)				
Positive	59 (79.7)	21 (75)	38 (82.6)	0.715
Negative	9 (12.2)	4 (14.3)	5 (10.9)	
Missing	6 (8.1)	3 (10.7)	3 (6.5)	
CD7 (*n*,%)				
Positive	22 (29.7)	9 (32.2)	13 (28.3)	0.670
Negative	45 (60.8)	16 (57.1)	29 (63)	
Missing	7 (9.5)	3 (10.7)	4 (8.7)	
CD25 (*n*,%)				
Positive	28 (37.8)	12 (42.9)	16 (34.8)	0.712
Negative	34 (45.9)	13 (46.4)	21 (45.6)	
Missing	12 (16.2)	3 (10.7)	9 (19.6)	
CD11c (*n*, %)				
Positive	54 (73)	19 (67.9)	35 (76.1)	
Negative	9 (12.2)	5 (17.8)	4 (8.7)	0.283
Missing	11 (14.8)	4 (14.3)	7 (15.2)	
TLS (*n*, %)				
Yes	10 (13.5)	4 (14.3)	6 (13)	1.00
No	64 (86.5)	24 (85.7)	40 (87)	
AKI (*n*, %)				
Yes	19 (25.7)	8 (28.6)	11 (23.9)	0.565
No	55 (74.3)	20 (71.4)	35 (76.1)	
DIC (*n*, %)				
Yes	45 (60.8)	18 (64.3)	27 (58.7)	0.633
No	29 (39.2)	10 (35.7)	19 (41.3)	
Treatment (*n*, %)				
Intensive	44 (59.5)	18 (64.3)	26 (56.5)	0.509
Non-intensive and palliative	30 (40.5)	10 (35.7)	20 (43.5)	
Allo-SCT (*n*, %)				
Yes	7 (9.5)	3 (10.7)	4 (8.7)	0.705
No	67 (90.5)	25 (89.3)	42 (91.3)	

Abbreviations: ECOG PS—Eastern Cooperative Oncology Group performance status, HCT-CI—Hematopoietic cell transplantation specific comorbidity index, CV—cardiovascular, WBC—white blood cell, LDH—lactate dehydrogenase, DIC—disseminated intravascular coagulation, TLS—tumor lysis syndrome, AKI—acute kidney injury, PB—peripheral blood, BM—bone marrow, SCT—stem cell transplantation.

**Table 2:** Factors influencing 15-, 30-, and 90-day mortality.

Parameters	15-Day Mortality	30-Day Mortality	90-Day Mortality
Age			
All	*p* = 0.375	*p* = 0.616	*p* = 0.345
LA	*p* = 0.264	*p* = 0.342	*p* = 0.120
non-LA	*p* = 0.739	*p* = 0.916	*p* = 0.939
ECOG PS ≥ 2			
All	***p* < 0.01**	***p* < 0.01**	***p* < 0.01**
LA	***p* = 0.01**	***p* = 0.003**	***p* < 0.01**
non-LA	***p* = 0.002**	***p* < 0.01**	***p* = 0.003**
HCT-CI > 2			
All	*p* = 0.871	*p* = 0.420	*p* = 0.809
LA	*p* = 0.407	*p* = 0.419	*p* = 0.492
non-LA	*p* = 0.301	*p* = 0.888	*p* = 0.357
≥2 comorbidities			
All	*p* = 0.462	*p* = 0.897	*p* = 0.297
LA	*p* = 0.455	*p* = 0.806	*p* = 0.904
non-LA	*p* = 0.657	*p* = 0.810	*p* = 0.156
CV comorbidity			
All	*p* = 0.416	*p* = 0.657	*p* = 0.182
LA	*p* = 0.456	*p* = 0.833	*p* = 0.913
non-LA	*p* = 0.652	*p* = 0.638	*p* = 0.093
EMD			
All	*p* = 0.205	*p* = 0.110	*p* = 0.259
LA	*p* = 0.205	*p* = 0.404	*p* = 0.398
non-LA	*p* = 0.654	*p* = 0.182	*p* = 0.479
WBC count			
All	*p* = 0.295	***p* = 0.032**	*p* = 0.071
LA	*p* = 0.669	*p* = 0.334	*p* = 0.109
non-LA	*p* = 0.072	*p* = 0.097	*p* = 0.540
Hb			
All	*p* = 0.444	*p* = 0.354	*p* = 0.263
LA	*p* = 0.641	*p* = 0.638	*p* = 0.813
non-LA	*p* = 0.421	*p* = 0.256	*p* = 0.118
PLT count			
All	*p* = 0.198	*p* = 0.225	*p* = 0.118
LA	*p* = 0.596	*p* = 0.935	*p* = 0.472
non-LA	*p* = 0.157	*p* = 0.055	*p* = 0.096
Monocytes PB			
All	*p* = 0.897	*p* = 0.506	*p* = 0.125
LA	***p* = 0.031**	*p* = 0.107	*p* = 0.285
Non-LA	*p* = 0.096	*p* = 0.088	***p* = 0.017**
LDH			
All	***p* < 0.01**	***p* < 0.01**	***p* < 0.01**
LA	***p* = 0.021**	*p* = 0.097	*p* = 0.068
non-LA	***p* < 0.01**	***p* < 0.01**	***p* < 0.01**
DIC			
All	***p* = 0.01**	***p* = 0.018**	*p* = 0.207
LA	*p* = 0.131	*p* = 0.116	*p* = 0.247
non-LA	***p* = 0.045**	*p* = 0.089	*p* = 0.520
TLS			
All	***p* = 0.01**	***p* = 0.002**	***p* = 0.017**
LA	***p* = 0.033**	***p* = 0.004**	***p* = 0.004**
non-LA	*p* = 0.120	*p* = 0.075	*p* = 0.382
AKI			
All	***p* = 0.001**	***p* = 0.001**	***p* = 0.015**
LA	***p* = 0.037**	***p* = 0.037**	***p* = 0.025**
Non-LA	***p* = 0.007**	***p* = 0.010**	*p* = 0.225
PB blast %			
All	*p* = 0.375	*p* = 0.072	***p* = 0.036**
LA	*p* = 0.150	*p* = 0.545	*p* = 0.943
non-LA	*p* = 0.056	***p* = 0.021**	***p* = 0.014**
BM blast %			
All	*p* = 0.679	*p* = 0.279	*p* = 0.238
LA	*p* = 0.261	*p* = 0.786	*p* = 0.950
non-LA	*p* = 0.138	*p* = 0.137	*p* = 0.175
Unfavorable cytogenetics			
All	*p* = 0.525	*p* = 0.848	*p* = 0.892
LA	*p* = 0.052	*p* = 0.284	*p* = 0.118
non-LA	*p* = 0.506	*p* = 0.812	*p* = 0.872
FLT3			
All	*p* = 0.069	*p* = 0.107	*p* = 0.144
LA	*p* = 0.065	*p* = 0.177	*p* = 0.083
non-LA	*p* = 0.521	*p* = 0.351	*p* = 0.573
NPM1			
All	*p* = 0.125	*p* = 0.329	*p* = 0.169
LA	*p* = 0.057	*p* = 0.099	*p* = 0.094
non-LA	*p* = 0.953	*p* = 0.201	*p* = 0.460
CD56			
All	*p* = 0.513	*p* = 0.769	*p* = 0.435
LA	*p* = 0.930	*p* = 0.936	*p* = 0.936
non-LA	*p* = 0.762	*p* = 0.783	*p* = 0.817
CD117			
All	*p* = 0.985	*p* = 0.668	*p* = 0.619
LA	*p* = 0.936	*p* = 0.936	*p* = 0.935
non-LA	*p* = 0.972	*p* = 0.779	*p* = 0.576
CD7			
All	*p* = 0.815	*p* = 0.568	*p* = 0.365
LA	*p* = 0.936	*p* = 0.936	*p* = 0.936
non-LA	*p* = 0.254	*p* = 0.459	*p* = 0.216
CD25			
All	*p* = 0.067	*p* = 0.145	*p* = 0.064
LA	*p* = 0.932	*p* = 0.934	*p* = 0.933
non-LA	***p* = 0.034**	*p* = 0.153	***p* = 0.048**
CD11c			
All	*p* = 0.470	*p* = 0.360	*p* = 0.876
LA	*p* = 0.057	*p* = 0.086	*p* = 0.170
non-LA	*p* = 0.547	*p* = 0.410	*p* = 0.884
Intensive induction therapy			
All	***p* < 0.01**	***p* < 0.01**	***p* < 0.01**
LA	***p* = 0.004**	***p* = 0.022**	***p* = 0.05**
non-LA	***p* = 0.007**	***p* = 0.002**	***p* = 0.008**

Abbreviations: ECOG PS—Eastern Cooperative Oncology Group performance status, HCT-CI—Hematopoietic cell transplantation specific comorbidity index, CV—cardiovascular, EMD—extramedullary disease, WBC—white blood cell, Hb—hemoglobin, PLT—platelet, LDH—lactate dehydrogenase, DIC—disseminated intravascular coagulation, TLS—tumor lysis syndrome, AKI—acute kidney injury, PB—peripheral blood, BM—bone marrow.

**Table 3:** Multivariate analysis of factors influencing overall survival (OS).

	HR	95% CI	*p* Value
WBC	0.998	0.994–1.002	*p* = 0.406
PB blast %	1.017	1.005–1.029	***p* = 0.004**
ECOG PS ≥ 2	4.588	1.675–12.569	***p* = 0.003**
LDH	1.000	1.000–1.000	*p* = 0.231
CD25	2.075	1.152–3.738	***p* = 0.015**
Intensive induction therapy	1.462	0.639–3.346	*p* = 0.369

Abbreviations: WBC—white blood cell, PB—peripheral blood, ECOG PS—Eastern Cooperative Oncology Group performance status, LDH—lactate dehydrogenase.

## Data Availability

The data that support the findings of this study are available on request from the corresponding author.

## Abbreviations

The following abbreviations are used in this manuscript:

AKI	Acute kidney injury
AML	Acute myeloid leukemia
BM	Bone marrow
DIC	Disseminated intravascular coagulation
ECOG PS	Eastern Cooperative Oncology Group performance status
ELN	European LeukemiaNet
EMD	Extramedullary disease
FLT3	FMS-like tyrosine kinase 3
Hb	Hemoglobin
HCT-CI	Hematopoietic cell transplantation specific comorbidity index
HL	Hyperleukocytosis
LA	Leukapheresis
LDH	Lactate dehydrogenase
OS	Overall survival
PB	Peripheral blood
Plt	Platelet
TLS	Tumor lysis syndrome
WBC	White blood cell
WHO	World Health Organization

## References

[1] Döhner H., Weisdorf D.J., Bloomfield C.D. (2015). Acute Myeloid Leukemia. N. Engl. J. Med..

[2] Shimony S., Stahl M., Stone R.M. (2025). Acute Myeloid Leukemia: 2025 Update on Diagnosis, Risk-stratification, and Management. Am. J. Hematol..

[3] Pastore F., Pastore A., Wittmann G., Hiddemann W., Spiekermann K. (2014). The Role of Therapeutic Leukapheresis in Hyperleukocytotic AML. PLoS ONE.

[4] Ganzel C., Becker J., Mintz P.D., Lazarus H.M., Rowe J.M. (2012). Hyperleukocytosis, Leukostasis and Leukapheresis: Practice Management. Blood Rev..

[5] Rinaldi I., Sutandyo N., Winston K. (2022). Comparison of Early Mortality between Leukapheresis and Non-Leukapheresis in Adult Acute Myeloid Leukemia Patients with Hyperleukocytosis: A Systematic Review and Meta-Analysis. Hematology.

[6] Röllig C., Ehninger G. (2015). How I Treat Hyperleukocytosis in Acute Myeloid Leukemia. Blood.

[7] Döhner H., Wei A.H., Appelbaum F.R., Craddock C., DiNardo C.D., Dombret H., Ebert B.L., Fenaux P., Godley L.A., Hasserjian R.P. (2022). Diagnosis and Management of AML in Adults: 2022 Recommendations from an International Expert Panel on Behalf of the ELN. Blood.

[8] Bewersdorf J.P., Zeidan A.M. (2020). Hyperleukocytosis and Leukostasis in Acute Myeloid Leukemia: Can a Better Understanding of the Underlying Molecular Pathophysiology Lead to Novel Treatments?. Cells.

[9] Shallis R.M., Stahl M., Bewersdorf J.P., Hendrickson J.E., Zeidan A.M. (2020). Leukocytapheresis for Patients with Acute Myeloid Leukemia Presenting with Hyperleukocytosis and Leukostasis: A Contemporary Appraisal of Outcomes and Benefits. Expert Rev. Hematol..

[10] Lichtman M.A., Rowe J.M. (1982). Hyperleukocytic Leukemias: Rheological, Clinical, and Therapeutic Considerations. Blood.

[11] Korkmaz S. (2018). The Management of Hyperleukocytosis in 2017: Do We Still Need Leukapheresis?. Transfus. Apher. Sci..

[12] Porcu P., Cripe L.D., Ng E.W., Bhatia S., Danielson C.M., Orazi A., McCarthy L.J. (2000). Hyperleukocytic Leukemias and Leukostasis: A Review of Pathophysiology, Clinical Presentation and Management. Leuk. Lymphoma.

[13] Stucki A., Rivier A.-S., Gikic M., Monai N., Schapira M., Spertini O. (2001). Endothelial Cell Activation by Myeloblasts: Molecular Mechanisms of Leukostasis and Leukemic Cell Dissemination. Blood.

[14] Hölig K., Moog R. (2012). Leukocyte Depletion by Therapeutic Leukocytapheresis in Patients with Leukemia. Transfus. Med. Hemother..

[15] Powell B.L., Gregory B.W., Evans J.K., White J.C., Lyerly E.S., Chorley H.M., Russell G.B., Capizzi R.L. (1991). Leukapheresis Induced Changes in Cell Cycle Distribution and Nucleoside Transporters in Patients with Untreated Acute Myeloid Leukemia. Leukemia.

[16] Padmanabhan A., Connelly-Smith L., Aqui N., Balogun R.A., Klingel R., Meyer E., Pham H.P., Schneiderman J., Witt V., Wu Y. (2019). Guidelines on the Use of Therapeutic Apheresis in Clinical Practice—Evidence-based Approach from the Writing Committee of the American Society for Apheresis: The Eighth Special Issue. J. Clin. Apher..

[17] Bug G., Anargyrou K., Tonn T., Bialleck H., Seifried E., Hoelzer D., Ottmann O.G. (2007). Impact of Leukapheresis on Early Death Rate in Adult Acute Myeloid Leukemia Presenting with Hyperleukocytosis. Transfusion.

[18] Nan X., Qin Q., Gentille C., Ensor J., Leveque C., Pingali S.R., Phan A.T., Rice L., Iyer S. (2017). Leukapheresis Reduces 4-Week Mortality in Acute Myeloid Leukemia Patients with Hyperleukocytosis—A Retrospective Study from a Tertiary Center. Leuk. Lymphoma.

[19] Lee H., Han J.H., Kim J.K., Yoo J., Cho H.S., Yoon J.-H., Cho B.S., Kim H.-J., Lim J., Jekarl D.W. (2023). Effectiveness of Leukapheresis on Early Survival in Acute Myeloid Leukemia: An Observational Propensity Score Matching Cohort Study. J. Clin. Apher..

[20] Stahl M., Shallis R.M., Wei W., Montesinos P., Lengline E., Neukirchen J., Bhatt V.R., Sekeres M.A., Fathi A.T., Konig H. (2020). Management of Hyperleukocytosis and Impact of Leukapheresis among Patients with Acute Myeloid Leukemia (AML) on Short- and Long-Term Clinical Outcomes: A Large, Retrospective, Multicenter, International Study. Leukemia.

[21] Rinaldi I., Sari R.M., Tedhy V.U., Winston K. (2021). Leukapheresis Does Not Improve Early Survival Outcome of Acute Myeloid Leukemia with Leukostasis Patients—A Dual-Center Retrospective Cohort Study. J. Blood Med..

[22] Choi M.H., Choe Y.H., Park Y., Nah H., Kim S., Jeong S.H., Kim H.O. (2018). The Effect of Therapeutic Leukapheresis on Early Complications and Outcomes in Patients with Acute Leukemia and Hyperleukocytosis: A Propensity Score-matched Study. Transfusion.

[23] Kuo K.H.M., Callum J.L., Panzarella T., Jacks L.M., Brandwein J., Crump M., Curtis J.E., Gupta V., Lipton J.H., Minden M.D. (2015). A Retrospective Observational Study of Leucoreductive Strategies to Manage Patients with Acute Myeloid Leukaemia Presenting with Hyperleucocytosis. Br. J. Haematol..

[24] Martínez-Cuadrón D., Montesinos P., Moscardo F., Lopez L., Martin G., Solves P., Boluda B., Pérez-Sirvent M., Vera B., Navarro I. (2013). Treatment with Leukapheresis in Patients Diagnosed with Hyperleukocytic Acute Myeloid Leukemia. Blood.

[25] Arber D.A., Orazi A., Hasserjian R., Thiele J., Borowitz M.J., Le Beau M.M., Bloomfield C.D., Cazzola M., Vardiman J.W. (2016). The 2016 Revision to the World Health Organization Classification of Myeloid Neoplasms and Acute Leukemia. Blood.

[26] Döhner H., Estey E., Grimwade D., Amadori S., Appelbaum F.R., Büchner T., Dombret H., Ebert B.L., Fenaux P., Larson R.A. (2017). Diagnosis and Management of AML in Adults: 2017 ELN Recommendations from an International Expert Panel. Blood.

[27] Oken M.M., Creech R.H., Tormey D.C., Horton J., Davis T.E., McFadden E.T., Carbone P.P. (1982). Toxicity and Response Criteria of the Eastern Cooperative Oncology Group. Am. J. Clin. Oncol..

[28] Sorror M.L., Storer B.E., Fathi A.T., Gerds A.T., Medeiros B.C., Shami P., Brunner A.M., Sekeres M.A., Mukherjee S., Peña E. (2017). Development and Validation of a Novel Acute Myeloid Leukemia–Composite Model to Estimate Risks of Mortality. JAMA Oncol..

[29] Suzuki K., Wada H., Imai H., Iba T., Thachil J., Toh C.-H. (2018). Subcommittee on Disseminated Intravascular Coagulation. A Re-Evaluation of the D-Dimer Cut-off Value for Making a Diagnosis According to the ISTH Overt-DIC Diagnostic Criteria: Communication from the SSC of the ISTH. J. Thromb. Haemost..

[30] Cairo M.S., Coiffier B., Reiter A., Younes A. (2010). TLS Expert Panel. Recommendations for the Evaluation of Risk and Prophylaxis of Tumour Lysis Syndrome (TLS) in Adults and Children with Malignant Diseases: An Expert TLS Panel Consensus. Br. J. Haematol..

[31] Pereira M., Rodrigues N., Godinho I., Gameiro J., Neves M., Gouveia J., Costa E Silva Z., Lopes J.A. (2017). Acute Kidney Injury in Patients with Severe Sepsis or Septic Shock: A Comparison between the “Risk, Injury, Failure, Loss of Kidney Function, End-Stage Kidney Disease” (RIFLE), Acute Kidney Injury Network (AKIN) and Kidney Disease: Improving Global Outcomes (KDIGO) Classifications. Clin. Kidney J..

[32] Pastore F., Pastore A., Rothenberg-Thurley M., Metzeler K.H., Ksienzyk B., Schneider S., Bohlander S.K., Braess J., Sauerland M.C., Görlich D. (2022). Molecular Profiling of Patients with Cytogenetically Normal Acute Myeloid Leukemia and Hyperleukocytosis. Cancer.

[33] Lin T.L., Pagano L. (2021). The Important Role of Intensive Induction Chemotherapy in the Treatment of Acute Myeloid Leukemia. Expert Rev. Hematol..

[34] Shallis R.M., Stahl M., Wei W., Montesinos P., Lengline E., Neukirchen J., Bhatt V.R., Sekeres M.A., Fathi A.T., Konig H. (2020). Patterns of Care and Clinical Outcomes of Patients with Newly Diagnosed Acute Myeloid Leukemia Presenting with Hyperleukocytosis Who Do Not Receive Intensive Chemotherapy. Leuk. Lymphoma.

[35] Porcu P., Farag S., Marcucci G., Cataland S.R., Kennedy M.S., Bissell M. (2002). Leukocytoreduction for Acute Leukemia. Ther. Apher..

[36] Zhang D., Zhu Y., Jin Y., Kaweme N.M., Dong Y. (2021). Leukapheresis and Hyperleukocytosis, Past and Future. Int. J. Gen. Med..

[37] Gönen M., Sun Z., Figueroa M.E., Patel J.P., Abdel-Wahab O., Racevskis J., Ketterling R.P., Fernandez H., Rowe J.M., Tallman M.S. (2012). CD25 Expression Status Improves Prognostic Risk Classification in AML Independent of Established Biomarkers: ECOG Phase 3 Trial, E1900. Blood.

[38] Li J., Ran Q., Xu B., Luo X., Song S., Xu D., Zhang X. (2020). Role of CD25 Expression on Prognosis of Acute Myeloid Leukemia: A Literature Review and Meta-Analysis. PLoS ONE.

[39] Farid K.M.N., Sauer T., Schmitt M., Müller-Tidow C., Schmitt A. (2023). Symptomatic Patients with Hyperleukocytic FLT3-ITD Mutated Acute Myeloid Leukemia Might Benefit from Leukapheresis. Cancers.

